# Integrated multi-omics analysis of metabolomics and proteomics uncovers dysregulated amino acid metabolism in HCC metastasis

**DOI:** 10.3389/fimmu.2026.1856643

**Published:** 2026-08-19

**Authors:** Jing Wu, Jin Wang, Zhen Yu, Rui Yang

**Affiliations:** 1 Department of Clinical Laboratory, Central Hospital, Tianjin University/Tianjin Third Central Hospital, Tianjin Key Laboratory of Extracorporeal Life Support for Critical Diseases, Tianjin Artificial Cell Engineering Technology Research Center, Tianjin Institute of Hepatobiliary Disease, Tianjin, China; 2 Department of Genetics, School of Basic Medical Sciences, Tianjin Medical University, Tianjin, China

**Keywords:** biomarker, hepatocellular carcinoma, metastasis, proteomics, untargeted metabolomics

## Abstract

**Background:**

Metastasis is the primary cause of treatment failure and adverse prognosis in hepatocellular carcinoma (HCC), and the molecular basis of HCC metastasis remains poorly defined. This work investigated the potential mechanisms underlying HCC metastasis through integrated multi-omics analysis of metabolomics and proteomics.

**Method:**

This retrospective study included 105 individuals with HCC, with comparative analysis between metastatic and non-metastatic cases. We further evaluated the effects of metastasis on serum metabolomics and proteomics in HCC patients.

**Result:**

Widespread disturbances in amino acid metabolism were identified via untargeted metabolomics in HCC patients with metastasis, closely governing inflammation-related metabolic remodeling and oxidative stress responses. Specifically, we identified 91 and 59 distinct differential metabolites capable of indicating HCC metastasis, with the screening criteria set as log_2_ fold change > 1.5, adjusted *P* value < 0.05, and VIP > 1.5 in positive and negative modes, respectively. The alanine, aspartate and glutamate metabolism pathway correlated with HCC-associated lung metastasis, while the gluconeogenesis pathway was linked to HCC-associated bone metastasis. Compared with HCC (non-metastatic hepatocellular carcinoma), the key molecular alterations in the multi-omics network of HCC_M (HCC with metastasis) are implicated in inflammatory metabolic reprogramming, oxidative stress response, gluconeogenesis, glycolysis, and the tricarboxylic acid (TCA) cycle. Twenty-five proteins, including PKM2, PERCK, ALDH2, CPS1, GLS1, GLUD1, GOT1, and SLC38A2, were identified as potential biomarkers for HCC metastasis.

**Conclusion:**

By integrating untargeted metabolomic and proteomic profiling, we identified distinct metabolic and proteomic changes linked to HCC metastasis. This work also characterized the pathological characteristics and core pathways underlying HCC metastasis, while identifying potential therapeutic candidates.

## 1 Introduction

Hepatocellular carcinoma (HCC), one of the most prevalent primary malignant tumors of the liver, is associated with high morbidity and mortality and thus imposes an increasingly heavy burden on global healthcare (1, 2). The prognosis of HCC patients remains dismal, with an overall 5-year survival rate of less than 20% (3). Presently, the conventional therapeutic regimen for HCC is early resection, local ablation therapy, interventional therapy, molecular targeted therapy and organ support therapy. Despite considerable advances made in treatment strategies for HCC in recent years (4, 5), the majority rate of HCC remains high. The reason is that the pathophysiology involved in HCC progression remains unclear and the patients are still diagnosed at intermediate-to-advanced stages with concomitant metastasis (6, 7). There is an increasing demand to refine the molecular classification of HCC, decipher its intricate molecular features, and develop high-precision detection approaches for multiple biomarkers. Such advances will facilitate early screening, risk warning and prognostic evaluation, allowing the implementation of timely, individualized and targeted therapeutic strategies for HCC patients.

Metabolomics is a research method that targets small-molecule metabolites with a relative molecular mass of less than 1 kDa in organisms (8). It employs mass spectrometry-based hyphenated techniques for the qualitative or quantitative analysis of endogenous small-molecule metabolites, thereby revealing alterations in internal metabolic pathways and exploring the relationship between endogenous small-molecule metabolites and the occurrence and progression of diseases (9). In recent years, significant progress has been made in the research on metabolic biomarkers of HCC using metabolomic technologies (10). Numerous studies have identified novel biomarkers associated with the progression, pathogenesis, and prognosis of HCC via metabolomic analyses and have also revealed distinct metabolic profiles specific to HCC (11, 12). However, most of these investigations merely adopted metabolomic profiling, along with differential screening and pathway enrichment analysis, to explore abnormal metabolic alterations during the initiation and progression of HCC. To date, research on screening for metastasis-related metabolic biomarkers of HCC using metabolomic technologies has yielded no substantial progress, and it remains challenging to fully elucidate the mechanisms underlying HCC metastasis. Multi-omics-based co-expression network analysis can yield valuable clues for revealing the regulatory events underlying HCC metastasis. Weighted Gene Co-expression Network Analysis (WGCNA) is a systems biology method in the field of bioinformatics for mining high-throughput gene expression data (13). Its core lies in constructing a co-expression correlation network among genes, identifying gene modules with coordinated expression patterns, and elucidating the associations between these modules and phenotypic traits (e.g., disease stage, metastatic potential) or clinical characteristics (14). With the rapid progress in multi-omics profiling technologies, developing a high-accuracy multimolecular biomarker system grounded on large-scale omics datasets and evidence-based medicine will become a key breakthrough for early screening, risk surveillance and prognostic evaluation of HCC.

For the first time, this study employed metabolomic techniques to detect the aggregation profiles of all small molecular metabolites in patients with HCC complicated by metastasis. Concurrently, WGCNA and multi-omics integrative analysis were performed on the same biological samples. The objectives are to investigate the variation patterns of metabolites *in vivo*, elucidate the metabolic mechanisms underlying HCC metastasis, and identify characteristic small-molecule metabolic biomarkers applicable for predicting HCC metastasis and investigating its pathogenesis, and thus providing mechanistic evidence and methodological references for the early detection, clinical prognosis assessment, and therapeutic target screening of HCC.

## 2 Methods

### 2.1 Study design

**Figure 1:**
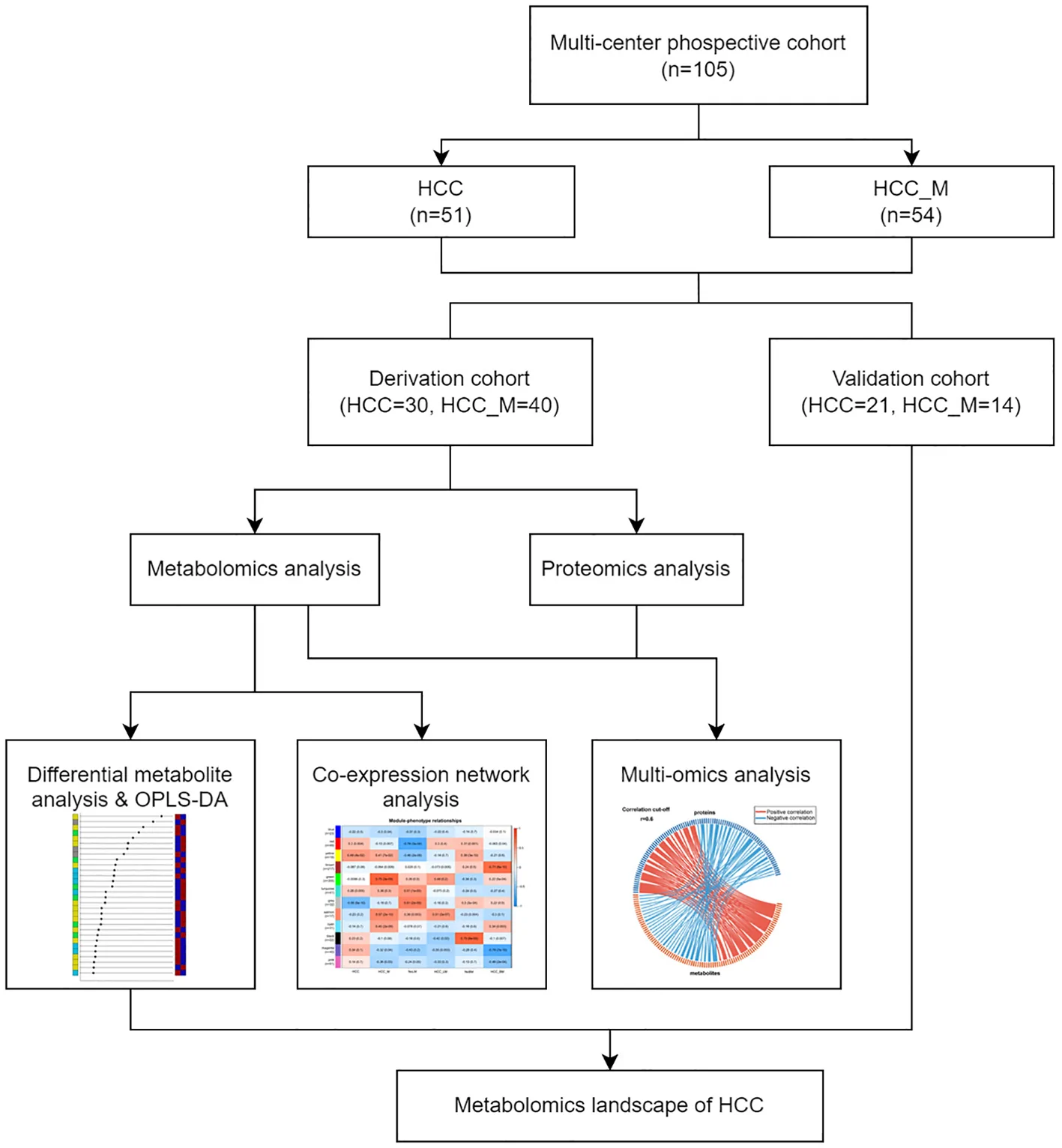
Overview of the study design and patient group allocation. One hundred and five subjects were studied, of whom 70 were randomly selected for proteomic and metabolomic analysis, and 35 were in the validation group. HCC_M, HCC patients with metastasis.

In this study, a total of 70 serum specimens were enrolled from Tianjin Medical University Cancer Institute and Hospital as the derivation cohort, comprising 30 from non-metastatic HCC patients (Edmondson grade I and II, HCC group) and 40 from HCC patients with lymph-node or distant organ metastasis (Edmondson grade III and IV, HCC_M group). For validation, an additional 35 clinical serum samples were allocated, consisting of 14 from metastatic (HCC_M) and 21 from non-metastatic (HCC) patients (**Figure 1**). The major inclusion requirements included: (1) definitive diagnosis of primary HCC confirmed by pathology or radiographic evaluation; (2) participant age of 18 years or above; (3) availability of complete clinical data; (4) All patients did not receive any preoperative anticancer treatments. Patient and pathological characteristics are summarized in **Table 1**. All specimens were preserved at -80 °C prior to subsequent experimentation. Differential screening and co-expression network profiling were applied to screen altered metabolites and dysregulated pathways closely correlated with the clinical and pathological features of HCC. In addition, integrated analysis of matched metabolomic and proteomic data from identical specimens was carried out to systematically characterize the overall molecular landscape underlying HCC progression. This study was approved by the Ethics Committee of Tianjin Third Central Hospital (affiliated to Tianjin University). Written informed consent was voluntarily provided by each participant prior to study enrollment.

**Table 1:** The clinical characteristics of participants included in the derivation group and validation group.

Characteristic	Derivation group			Validation group		
	HCC	HCC_M	*P* value	HCC	HCC_M	*P* value
n	30	40		21	14	
Age (mean ± SD)	60.5 ± 11.0	57.2 ± 10.1	0.400	60.7 ± 12.0	58.1 ± 10.9	0.573
Male (%)	28 (93.3%)	37 (92.5%)	0.893	20 (95.2%)	13 (92.9%)	0.766
Tumor size			0.003			0.042
<5cm	12 (40.0%)	4 (10.0%)		10 (47.6%)	2 (14.3%)	
≥5cm	18 (60.0%)	36 (90.0%)		11 (52.4%)	12 (85.7%)	
CEA (%)			0.139			0.886
<5μg/L	21 (70%)	21 (52.5%)		13 (61.9%)	9 (64.3%)	
≥5μg/L	9 (30%)	19 (47.5%)		8 (38.1%)	5 (35.7%)	
AFP (%)			0.436			0.036
<400ng/mL	20 (66.7%)	23 (57.5%)		15 (71.4%)	5 (35.7%)	
≥400ng/mL	10 (33.3%)	17 (42.5%)		6 (28.6%)	9 (64.3%)	
Tumor Invasion (T stage)			0.000			0.000
T1 + T2	30 (100%)	0 (0%)		21 (100%)	0 (0%)	
T3 + T4	0 (0%)	40 (100%)		0 (0%)	14 (100%)	
Lymph node metastasis (N stage)			0.000			0.000
N0	30 (100%)	0 (0%)		21 (100%)	0 (0%)	
N1-N3	0 (0%)	40 (100%)		0 (0%)	14 (100%)	
Distant metastasis (M stage)			0.000			0.000
M0	30 (100%)	0 (0%)		21 (100%)	0 (0%)	
M1	0 (0%)	40 (100%)		0 (0%)	14 (100%)	
HBsAg			0.375			0.490
Negative	7 (23.3%)	6 (15%)		5 (23.8%)	2 (14.3%)	
Positive	23 (76.7%)	34 (85%)		16 (76.2%)	12 (85.7%)	
Edmondson grade (%)			0.000			0.000
I-II	30 (100%)	0 (0%)		21 (100%)	0 (0%)	
III-IV	0 (0%)	40 (100%)		0 (0%)	14 (100%)	

FDR-adjusted P < 0.05.

### 2.2 Preparation of serum specimens for metabolomic profiling

Briefly, 100 μL of peripheral blood was transferred into Eppendorf tubes, and samples were resuspended in pre-cooled 80% methanol through vigorous vortexing. After 5 minutes of ice incubation, the mixture was centrifuged at 15,000 g and 4 °C for 20 min. The collected supernatant was diluted with LC-MS grade water to reach a final methanol concentration of 53%. Thereafter, the diluted solution was transferred into new sterile Eppendorf tubes and subjected to a second centrifugation under the same centrifugal conditions. The final supernatant was harvested and cryopreserved at -80 °C for subsequent LC-MS/MS metabolomic profiling (15).

### 2.3 Metabolomic LC-MS/MS analysis

Chromatographic and mass spectrometric measurements were carried out on a Vanquish UHPLC platform (Thermo Fisher Scientific, Germany) coupled with high-resolution Orbitrap mass analyzers, including Q Exactive HF, Q Exactive HF-X, Exploris 120 and Exploris 480 instruments (Thermo Fisher Scientific, Germany). Analytes were separated using a Hypersil Gold column (100 × 2.1 mm, 1.9 μm) with a 12-minute linear elution gradient at a constant flow rate of 0.2 mL/min. Mobile phase A consisted of 0.1% formic acid in ultrapure water, while pure methanol was adopted as mobile phase B for both positive and negative ionization modes. The gradient elution program was configured as follows: maintained at 2% B for the initial 1.5 min; linearly increased from 2% to 85% B within 3 min; held at 85%-100% B for 10 min; returned to 2% B at 10.1 min and finally stabilized at 2% B until the 12-min endpoint. For Q Exactive HF acquisition, the mass spectrometer was operated under dual positive and negative ion modes. The electrospray voltage was set to 3.5 kV, with a capillary temperature of 320 °C. The sheath gas pressure was adjusted to 35 psi, and the auxiliary gas flow was maintained at 10 L/min. The S-lens RF value was fixed at 60, and the auxiliary gas heater was kept at 350 °C throughout the detection process.

### 2.4 Processing and analysis of metabolomic datasets

Raw UHPLC-MS/MS datasets were imported into the XCMS platform for systematic processing, including peak detection, alignment and quantitative calculation of individual metabolites. Metabolite annotation was accomplished using NovoMetDB, an experimentally validated MS² spectral database (NovoPro Bioscience). The annotation pipeline applied stringent matching criteria: precursor mass error < 10 ppm, MS² spectral similarity score > 0.7, consideration of common adduct ions, and automatic cross-referencing to HMDB and KEGG identifiers upon successful spectral matching. Background interference ions were excluded according to blank control information. Original quantitative signals were then normalized to calculate relative peak areas with the following formula: Relative peak area = Individual raw quantification value/(Total sample quantification value/Total quantitative value of QC1). Metabolites with a coefficient of variation (CV) exceeding 30% in quality control specimens were filtered out to ensure data stability. For multiple hypothesis testing, the Benjamini-Hochberg false discovery rate (FDR) correction was applied, with a significance threshold set at FDR-adjusted P < 0.05. Ultimately, reliable metabolite identification and relative quantification outcomes were acquired. All computational workflows were conducted on a Linux system (CentOS 6.6), with R and Python adopted for program operation and statistical calculation. Public online databases, such as HMDB, KEGG and MetaboAnalyst, were comprehensively utilized to annotate metabolite functions and enrich aberrant metabolic pathways.

### 2.5 Total protein extraction and trypsin treatment

BioRAD Proteominer beads and serum specimens were added to 1.5 mL centrifuge tubes, followed by incubation on a vertical rotary shaker for 2 hours to ensure sufficient mixing. After incubation, the mixture was centrifuged at 10,000 g and 4 °C for 5 minutes, and the collected beads were thoroughly washed with wash buffer through repeated rinsing steps. Subsequently, 0.4 mL of 1% trifluoroacetic acid (TFA) was added to the washed beads, and the mixture was vortexed repeatedly for 10 minutes to elute the target proteins. The supernatant was carefully collected, and this elution process was repeated twice to maximize protein recovery. The combined supernatant was freeze-dried into a dry powder using a freeze concentrator. The resulting pellet was resuspended in a dissolution buffer consisting of 6 M urea and 100 mM triethylammonium bicarbonate (TEAB, pH = 8.5) until completely dissolved. To reduce disulfide bonds, 1 M DL-dithiothreitol (DTT) was added to the solution, which was then incubated at 56 °C for 1 hour. Thereafter, the sample was alkylated with an adequate amount of iodoacetamide (IAM) at room temperature in the dark for 1 hour, followed by a 2-minute ice bath to terminate the alkylation reaction.

100 μL of each protein sample was aliquoted, and its volume was adjusted to 100 μL with DB lysis buffer (containing 6 M Urea and 100 mM TEAB, pH 8.5). Subsequently, trypsin and 100 mM TEAB buffer were added to the sample, which was thoroughly mixed and then incubated at 37 °C for 4 hours for enzymatic digestion. After digestion, formic acid was added to the sample to adjust the pH to below 3, followed by centrifugation at 12,000 g for 5 minutes at room temperature. The resulting supernatant was slowly loaded onto a C18 desalting column, which was then washed 3 times with washing buffer (composed of 0.1% formic acid and 3% acetonitrile). Finally, elution buffer (containing 0.1% formic acid and 70% acetonitrile) was added, the elevator of each sample was collected, and lyophilized to obtain dried peptide samples for subsequent proteomic detection.

### 2.6 Proteomics LC-MS/MS analysis

Mobile phases A and B were prepared prior to detection: mobile phase A consisted of 99.9% water and 0.1% formic acid, while mobile phase B was composed of 80% acetonitrile and 0.1% formic acid. The lyophilized peptide powder was reconstituted with 10 μL of mobile phase A, followed by centrifugation at 14,000 g and 4 °C for 20 minutes. A 200-ng aliquot of the resulting supernatant was injected into the system for liquid chromatography-mass spectrometry (LC-MS) detection. Chromatographic separation was performed using a Vanquish Neo upgraded UHPLC system (Thermo Fisher). The system was equipped with a C18 pre-column (catalog No. 174500, 5 mm × 300 μm, 5 μm, Thermo Fisher) maintained at 50 °C in a column oven, and a C18 analytical column (catalog No. ES906, PepMap™ Neo UHPLC, 150 μm × 15 cm, 2 μm, Thermo Fisher). The elution conditions for liquid chromatography are detailed in **Table 1**. Mass spectrometric detection was carried out using a Thermo Orbitrap Astral mass spectrometer equipped with an Easy-spray electrospray ionization (ESI) source. The ion spray voltage was set to 2.0 kV, and the ion transfer tube temperature was adjusted to 290 °C. The mass spectrometer was operated in data-independent acquisition (DIA) mode, with a full primary mass spectrometry scanning range of m/z 380–980. The primary MS resolution was set to 240,000 (at 200 m/z), and the automatic gain control (AGC) was set to 500%. The parent ion window size was 2-Th, with 300 DIA windows configured. The normalized collision energy (NCE) was set to 25%, the secondary mass spectrometry (MS/MS) acquisition range was m/z 150–2000, the secondary ion resolution of the Orbitrap Astral was set to 80,000, and the maximum injection time was 3 ms. All mass spectrometric detection data were collected as raw files (. raw format).

### 2.7 Proteomics data analysis

The raw.raw files were searched and analyzed using DIA-NN library search software. The mass deviations of both precursor ions and fragment ions were automatically detected and corrected by the software. For protein modification settings, carbamidomethylation of cysteine (C) was set as a fixed modification, while methionine (M) excision at the N-terminus was regarded as a variable modification. A maximum of 2 missed cleavage sites were permitted during the search process. To ensure the reliability and accuracy of the analysis results, the DIA-NN software further filtered the search outcomes, retaining only peptides with a Global.Q.Value < 0.01 and proteins with a PG.Q.Value < 0.01. Differentially expressed proteins (DEPs) were defined as those with |log_2_ fold change| > 1.5 and FDR-adjusted P < 0.05 (Benjamini-Hochberg method).

Functional analysis of Gene Ontology (GO) and InterPro (IPR) was performed via the InterProScan program, with searches conducted against a non-redundant protein database encompassing Pfam, PRINTS, ProDom, SMART, ProSite, and PANTHER (16). Meanwhile, the Clusters of Orthologous Groups (COG) and Kyoto Encyclopedia of Genes and Genomes (KEGG) databases were employed to analyze the protein families and metabolic pathways associated with the identified proteins. Differentially expressed proteins (DEPs) were utilized for a series of analytical processes, including volcanic map analysis, cluster heatmap analysis, as well as GO, IPR, and KEGG enrichment analyses (17).

### 2.8 Metabolite co-expression network analysis

WGCNA (Weighted Gene Co-Expression Network Analysis) was applied to identify metabolite co-expression modules based on untargeted metabolomic data (18). Dimensionality reduction of the dataset was realized by clustering metabolites with high correlation into distinct modules, where module membership (defined as kME) was used as a quantitative indicator to assess the association between each individual metabolite and the module eigengenes. To screen out metabolite modules closely related to the target clinical phenotype, correlation analyses were carried out between each module and the clinical characteristics of the samples.

The co-expression network was constructed using the blockwiseModules() function embedded in the WGCNA package, with the following parameter settings: the soft-thresholding power (β) was set to 3 (determined in accordance with the scale-free topology criterion), mergeCutHeight was 0.05, deepSplit was set to 4, minModuleSize was 10, mergePercent was 25%, and threshPercent was 50%; all other parameters were kept at their default values.

### 2.9 Multi-omics data integration

To integrate metabolomic and proteomic data for comprehensive analysis, sparse generalized canonical correlation discriminant analysis was conducted using the Data Integration Analysis for Biomarker Discovery using Latent cOmponents (DIABLO) framework (19), which is embedded in the R package mixOmics (20). This analytical method adopts a generalized supervised partial least squares (PLS) approach to integrate multiple types of omics data obtained from the same biological samples, thereby enabling the joint identification of key omics features across different datasets. Prior to the integration process using the DIABLO framework, the normalized metabolomic and proteomic datasets were preprocessed through log transformation to ensure data normality and improve the reliability of subsequent integration analysis.

### 2.10 Statistical analysis and reproducibility

#### 2.10.1 Sample size

Sample size was determined by available biobanked serum specimens meeting inclusion criteria. *Post-hoc* power analysis using the pwr package (R v4.3.1) showed the derivation cohort (n = 70) achieved >85% power to detect metabolite differences at |log_2_FC| > 1.5 and α = 0.05 (two-sided).

#### 2.10.2 Batch-effect correction

Pooled QC samples were injected every 10 samples to monitor run-to-run variation. Metabolites with QC CV > 30% were excluded, and total sum normalization was applied to correct intensity drift. Analytical stability was confirmed by Pearson r > 0.90 among QCs and tight PCA clustering (**Supplementary Figure 1**).

#### 2.10.3 WGCNA parameters

Weighted metabolite co-expression networks were built using WGCNA (v1.72) with β = 3 (scale-free R² > 0.80) and biweight midcorrelation. Module detection used mergeCutHeight = 0.05, deepSplit = 4, minModuleSize = 10, mergePercent = 25%, and threshPercent = 50%.

#### 2.10.4 DIABLO parameters

Multi-omics integration used mixOmics (v6.24.0) with ncomp = 3 determined by 10-fold cross-validation (10× repeats). The metabolite-protein design matrix weight was 0.1, and variable selection was optimized by tune.block.splsda() to minimize balanced classification error.

#### 2.10.5 Missing value imputation

For metabolomics, features with <50% non-zero detection were removed and remaining missing values were imputed with the per-metabolite minimum. For proteomics, proteins with >50% missing values were excluded (remaining values handled by DIA-NN). Both datasets underwent log_2_ transformation and unit-variance scaling prior to integration.

### 2.11 Statistical analysis

Unless stated otherwise, the measurement results of categorical variables were expressed as counts (percentages), while normally distributed variables were presented as mean ± standard deviation (SD), and non-normally distributed variables were reported as median (interquartile range, IQR). For intergroup comparisons: if continuous variables in both groups followed a normal distribution (Shapiro-Wilk test, P > 0.05), Welch’s t-test was applied to assess intergroup differences; for non-normally distributed data, the nonparametric Mann-Whitney U test was utilized for intergroup comparison. To control Type I error in multiple testing scenarios, the false discovery rate (FDR) was adjusted using the Benjamini-Hochberg procedure, with a significance threshold set at FDR-adjusted P < 0.05. Only ion features with non-zero measurement values accounting for more than 50% of the total were selected for subsequent analysis. Missing values in the metabolomic dataset were addressed using the minimum value imputation method. Following sum normalization, log10 transformation was performed on metabolite abundance values to ensure data normality. The chi-square test (χ² test) was adopted for intergroup comparisons of categorical variables. The criteria for defining statistical significance and differentially expressed metabolites (DEMs) were set as follows: log2 fold change (FC) > 1.5 and adjusted P-value < 0.05.

Statistical analyses were conducted using R software. Principal component analysis (PCA) was performed with the “PCA” function embedded in the FactoMineR package; orthogonal partial least squares-discriminant analysis (OPLS-DA), pathway analysis, and data visualization were all carried out via MetaboAnalyst 5.0 (http://www.metaboanalyst.ca/MetaboAnalyst/).

## 3 Results

### 3.1 Baseline clinical characteristics of study participants

This study recruited 105 participants overall, consisting of 51 patients with non-metastatic HCC (HCC) and 54 individuals with metastatic HCC (HCC_M). All subjects were randomly assigned to a derivation cohort (30 non-metastatic and 40 metastatic HCC cases) and a validation cohort (21 non-metastatic and 14 metastatic HCC cases), as illustrated in **Figure 1**. Clinical profiles of HCC individuals across the derivation and validation cohorts are summarized in **Table 1**. No significant inter-cohort differences were observed in sex, age, or routine laboratory parameters (P > 0.05). Baseline features of patients within the derivation cohort are further listed in **Supplementary Table 1**. In this primary cohort, age and gender were comparable between non-metastatic HCC and metastatic HCC_M subgroups, with all comparisons yielding P > 0.05.

### 3.2 Non-targeted metabolome analysis of serum samples from HCC cases

To guarantee the robustness of untargeted metabolomics data, overlapping profiles of response intensity and retention time for all chromatographic peaks in the total ion chromatogram (TIC) verified the stability of the detection instrument (**Supplementary Figure 1A**). Pearson correlation coefficients between quality control (QC) samples were calculated based on the relative quantitative values of metabolites (21). Stronger correlations across QC samples, reflected by correlation coefficients closer to 1, reflected favorable experimental stability and high-quality metabolomic measurements (**Supplementary Figure 1B**). In addition, PCA results revealed tight clustering of QC samples under both positive and negative ion conditions, confirming satisfactory technical repeatability for the overall LC-MS workflow (**Supplementary Figure 1C**).

**Figure 2:**
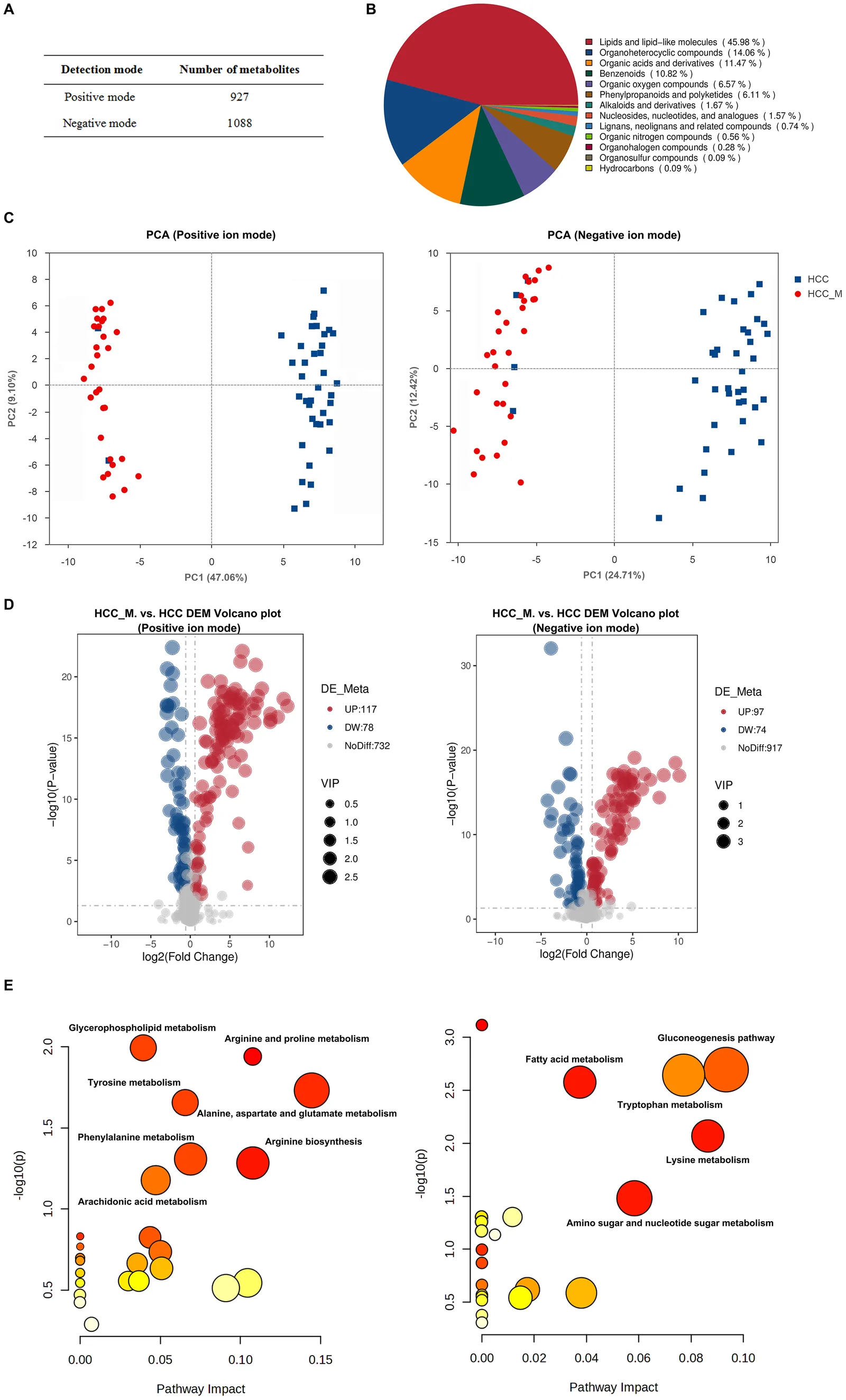
Comprehensive analysis of untargeted metabolomics of serum from HCC patients and HCC_M patients. **(A)** The number of metabolites detected in positive and negative modes. **(B)** The proportion of the identified metabolites in each chemical classification. **(C)** Principal component analysis (PCA) of the identified metabolites showing that HCC_M patients differed from HCC patients in both positive ion mode (left) and negative ion mode (right). **(D)** The volcano plot of differentially expressed metabolites (DEMs) between HCC_M patients and HCC patients in positive ion mode (left) and negative ion mode (right). The metabolites whose expressions increased in HCC_M patients are shown in red and those whose expressions decreased are shown in blue. **(E)** Pathway analysis of upregulated (left) and downregulated (right) metabolites.

This study identified 2015 distinct metabolites in total (**Figure 2A**), which were mainly classified into lipids and lipid-like molecules, organoheterocyclic compounds, organic acids and their derivatives, benzenoids, and other chemical categories (**Figure 2B**). The PCA score plot constructed using the identified metabolites from non-targeted metabolomic profiling revealed clear separation between HCC patients and HCC with metastasis patients in both positive and negative ion modes (**Figure 2C**). Differential metabolite screening was conducted using the following criteria: |log_2_ fold change| > 1.5, VIP score > 1.5, and Benjamini--Hochberg FDR-adjusted P < 0.05. Based on positive and negative ion detection modes, 195 significantly altered metabolites (116 elevated, 79 depressed) and 171 differential metabolites (97 elevated, 74 depressed) were separately screened in metastatic HCC patients (**Figure 2D**). Further details regarding the differentially expressed metabolites (DEMs) were presented in **Supplementary Table 2** and **Supplementary Table 3**. Integrated pathway enrichment analysis of collectively upregulated metabolites from both ion modes revealed prominent enrichment in amino acid metabolic cascades. Key enriched routes included alanine, aspartate and glutamate metabolism, arginine and proline metabolism, as well as arginine biosynthesis (**Figures 2E**, left). Depressed metabolites detected in the HCC_M group were predominantly involved in gluconeogenesis pathway, tryptophan metabolism and lysine metabolism (**Figures 2E**, right).

### 3.3 Potential diagnostic metabolite markers in metastatic HCC

**Figure 3:**
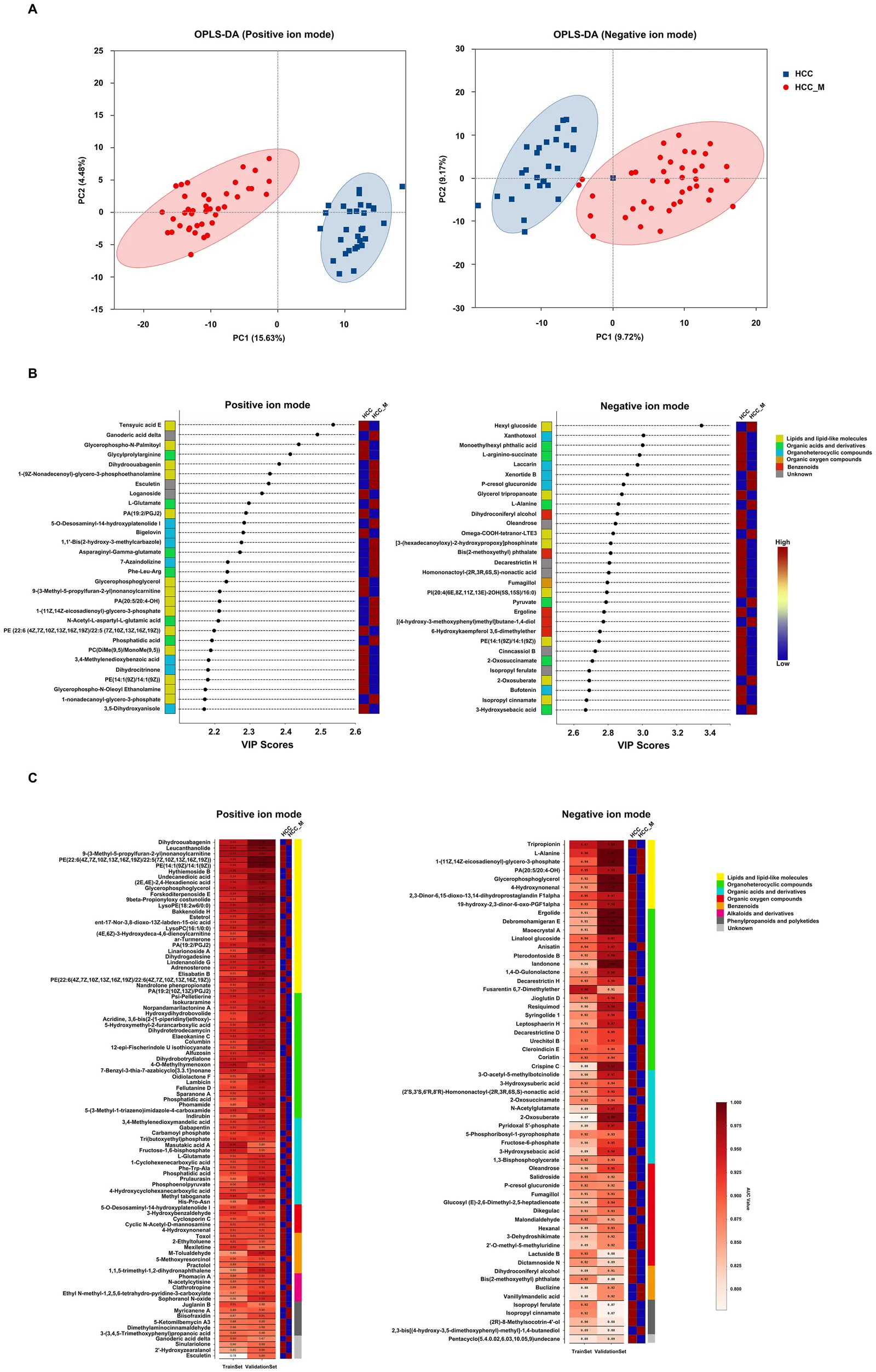
Dysregulated metabolites in HCC patients with metastasis. **(A)** Orthogonal partial least-squares discriminant analysis (OPLS-DA) of serum metabolism showing that HCC_M patients substantially differed from HCC patients in both positive (left) and negative ion modes (right). **(B)** The top 30 metabolites with high discriminatory accuracy ranked by variable importance in projection (VIP) score in positive ion mode (left) and negative ion mode (right). **(C)** The area under the receiver operating characteristic curve (AUROC) to assess the diagnostic accuracy of 91 metabolites (left) and 59 metabolites (right) in differentiating HCC_M patients from HCC patients in the derivation and validation groups.

OPLS-DA analysis showed obvious separation between HCC and metastatic HCC patients under both ion modes, suggesting distinct differences in their untargeted metabolomic profiles (**Figure 3A**). To evaluate potential overfitting of the OPLS-DA model, permutation testing was performed with 200 iterations under both ion modes. The results demonstrated that all permuted Q² values were lower than the original Q² values, with R²Y intercepts of 0.785 (positive) and 0.818 (negative), and Q² intercepts of 0.645 (positive) and 0.646 (negative) (**Supplementary Figure 2**). The Q² values exceeding 0.5 and the acceptable R²Y-Q² gaps (Δ < 0.2) indicated satisfactory model robustness without severe overfitting. The predictive performance was further corroborated by independent external validation (**Figure 3C**). The top 30 metabolites with the highest Variable Importance in Projection (VIP) scores were presented in **Figure 3B**, indicating their strong discriminatory potential for differentiating HCC_M patients from HCC patients. To identify candidate metabolite biomarkers for diagnosing metastatic HCC, we identified 91 and 59 significant DEMs in both ion modes, respectively. These DEMs met the predefined thresholds of |log_2_ fold change| > 1.5, VIP score > 1.5, and FDR-adjusted P < 0.05 (Benjamini-Hochberg method). (**Supplementary Figure 3**). To evaluate the diagnostic accuracy of candidate metabolite biomarkers, we calculated the area under the receiver operating characteristic curve (AUROC) for each potential marker. The results demonstrated that 150 metabolites-including the 91 and 59 significant DEMs identified in the positive and negative ion modes respectively exhibited AUROCs ranging from 0.78 to 0.98 (**Figure 3C**). Consistent outcomes were observed in the validation group, where the AUROC values spanned from 0.87 to 1.00. Within the 150 metabolites, some lipid molecules, such as dihydroouabagenin, PE(22:6(4Z,7Z,10Z,13Z,16Z,19Z)/22:5(7Z,10Z,13Z,16Z,19Z)), undecanedioic acid and PE(14:1(9Z)/14:1(9Z)), exert regulatory effects on energy metabolic reprogramming. Additionally, glycerophosphoglycerol plays a crucial role in glycerophospholipid metabolism and mitochondrial cardiolipin homeostasis.

### 3.4 Construction of HCC with metastasis metabolite co-expression network

**Figure 4:**
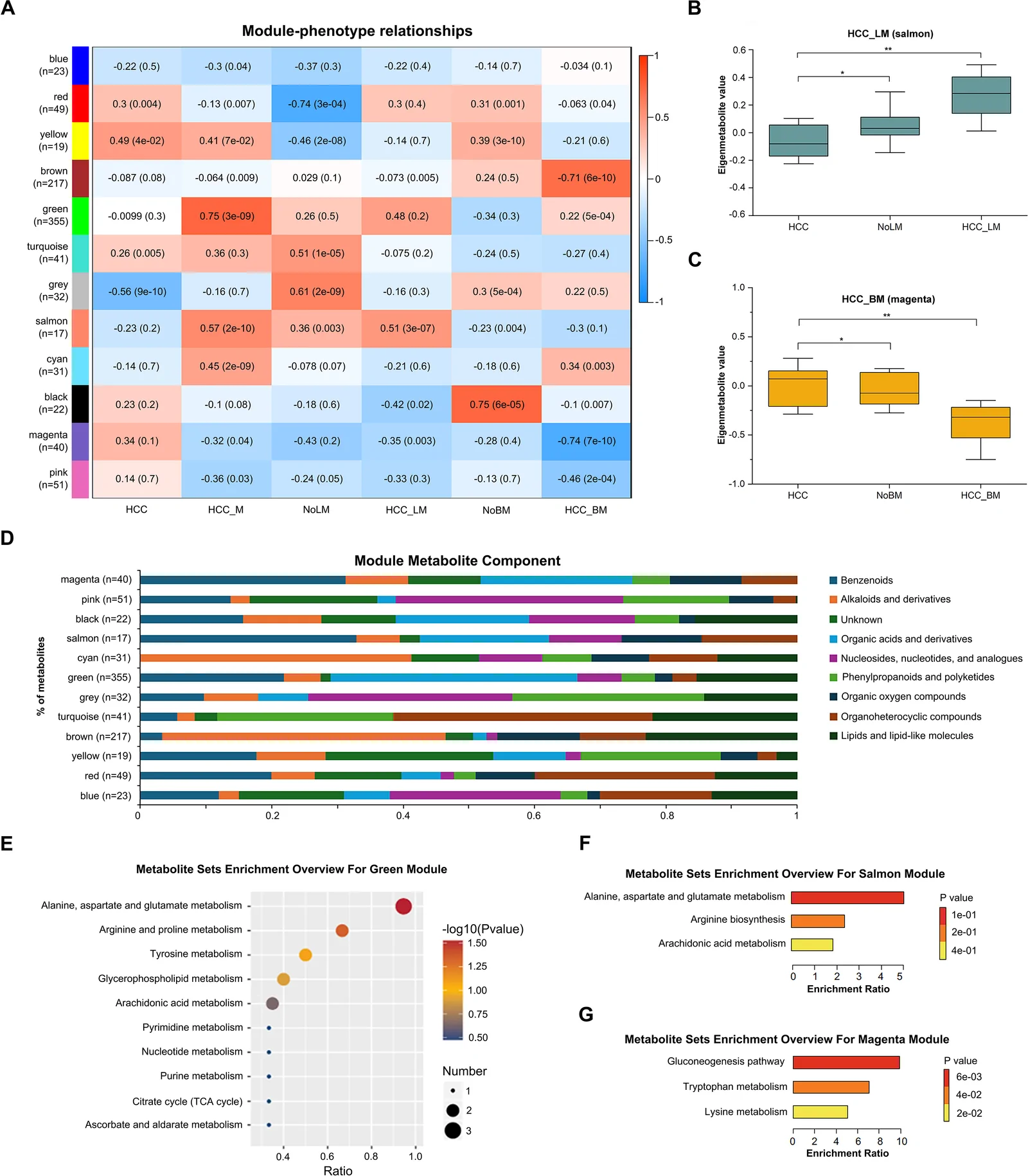
The coexpression network of serum metabolites constructed by weighted gene coexpression network analysis. **(A)** A Heatmap representation of the correlation between module eigenmetabolites and different phenotypes of HCC and HCC_M, HCC-associated lung metastasis (HCC_LM), and HCC-associated bone metastasis (HCC_BM). **(B)** Synthetic eigenmetabolite analysis of the salmon module, which is highly correlated with HCC_LM, except the green module. **(C)** Synthetic eigenmetabolite analysis for magenta module, which is highly correlated with HCC_BM, except for green module. **(D)** The distribution of metabolite members in each module according to the metabolite categories in **Figure 2B**. **(E)** Pathway analysis of metabolites in green module. **(F)** Pathway analysis of metabolites in salmon module. **(G)** Pathway analysis of metabolites in magenta module. *P < 0.05; **P < 0.01 (Student's t-test).

WGCNA was utilized to establish a metabolite co-expression network, which systematically captured the correlation patterns across all detected metabolites. Totally, 12 distinct metabolite modules characterized by robust intra-module co-expression were successfully identified. The number of metabolites contained in each module differed significantly, with the salmon module harboring 17 metabolites and the green module containing up to 355 metabolites. To pinpoint functionally relevant metabolic signatures, we subsequently performed module-trait correlation analysis to detect modules that exhibited significant associations with key clinical phenotypes relevant to HCC. Our results demonstrated a strong correlation between the green module and HCC_M, with a correlation coefficient (R) of 0.75 and a statistical significance (P value) of 3e-09 (**Figure 4A**). Besides the green module, the salmon module was found to be associated with HCC-associated lung metastasis (HCC_LM, R = 0.51, *P* value = 3e-07), and magenta module was correlated with HCC-associated bone metastasis (HCC_BM, R = -0.74, *P* value = 7e-10). The module eigenmetabolite level of the salmon module was significantly elevated across the HCC, NoLM and HCC_LM phenotypes (**Figure 4B**). Likewise, the module eigenmetabolite level of magenta module significantly reduced among the HCC, NoBM and HCC_BM phenotypes (**Figure 4C**). The types and classifications of metabolites in each module were depicted in **Figure 4D**. Organic acids, benzenoids and their derivatives constituted dominant metabolite classes in green, salmon, and magenta modules. Lipids and lipid-like molecules were detected in the green module but not in salmon and magenta modules, which included metabolites of organoheterocyclic compounds and organic oxygen compounds. Metabolic pathway enrichment analysis demonstrated that metabolites in the green module were mainly distributed in alanine, aspartate and glutamate metabolism, arginine and proline metabolism, tyrosine and glycerophospholipid metabolism (**Figure 4E**). The salmon module, which was positively correlated with HCC_LM, was significantly enriched in alanine, aspartate and glutamate metabolism (**Figure 4F**). On the contrary, metabolites in the magenta module that exhibited a negative association with HCC_BM were primarily involved in gluconeogenesis pathway, as well as tryptophan metabolism (**Figure 4G**).

### 3.5 Integrative analysis of untargeted metabolome and proteome profiles

**Figure 5:**
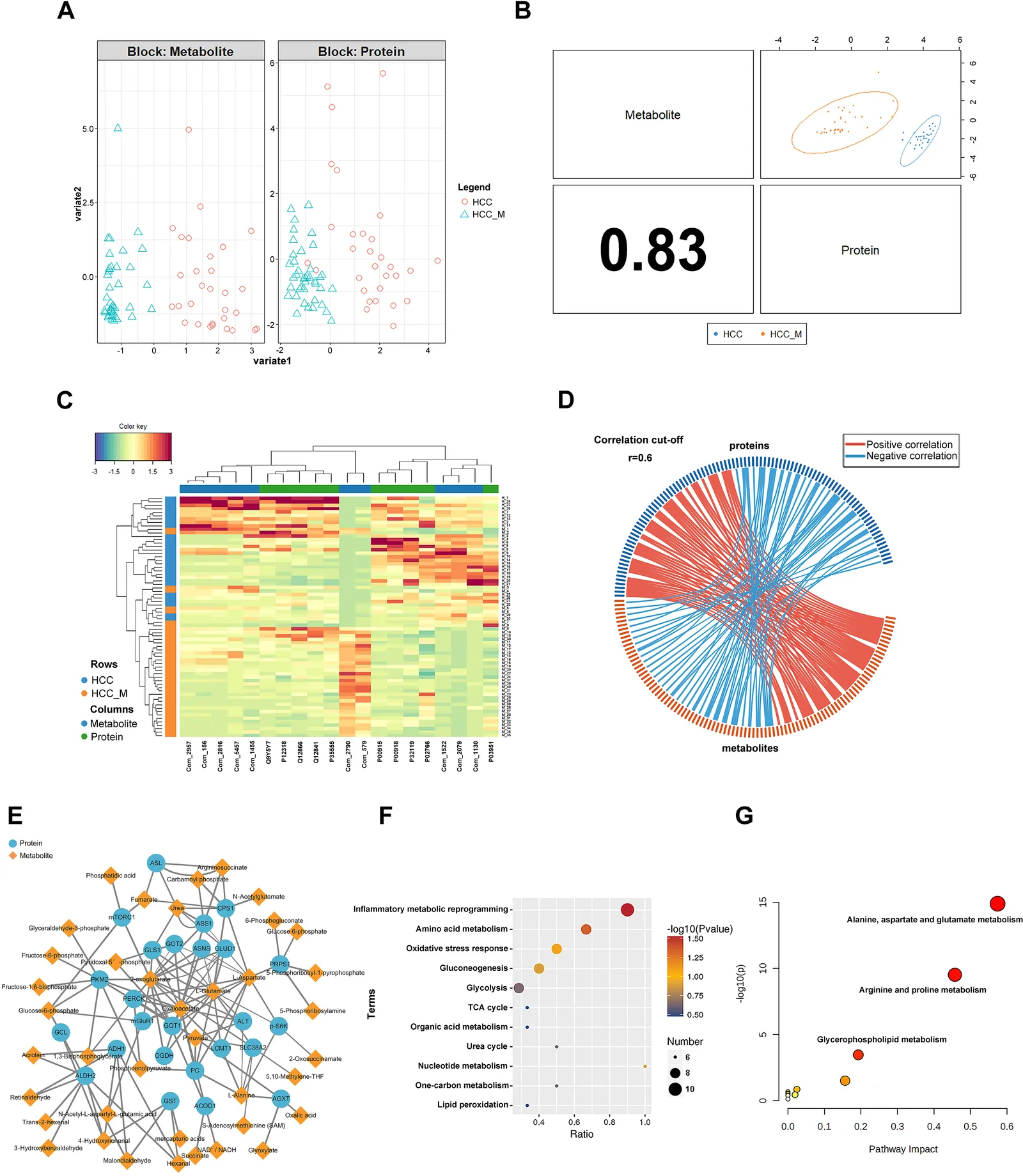
Integrative network analysis of proteomics and untargeted metabolomics data. **(A)** The considerable discrimination between HCC patients (HCC) and HCC patients with metastasis (HCC_M) in both untargeted metabolomics (left) and proteomics (right) data. **(B)** The Pearson correlation between proteomics and untargeted metabolomics data of their first component. **(C)** Integrated clustering heatmap of metabolomic and proteomic profiles in HCC and HCC_M samples. **(D)** Circos plot of close correlations (Pearson coefficient cut-offs set at ≥ 0.6 or ≤ -0.6) between proteomics and untargeted metabolomics data. **(E)** The network of key features across proteomics and untargeted metabolomics. The thickness of the edge represents the correlation coefficient. **(F)** GO terms enriched by the key proteins. **(G)** Pathway analysis of the key metabolites.

Using paired proteomic and metabolomic data derived from the same batch of biological samples, we conducted an integrative multi-omics analysis to establish a holistic molecular profile of HCC metastasis and elucidate the interplay between the proteome and metabolome. Distinct clustering of HCC_M patients and HCC patients was observed in the sample plot generated by the DIABLO model, indicating substantial shifts in both proteomic and metabolomic profiles associated with the metastasis of HCC (**Figure 5A**). A high correlation was observed between the latent components derived from the two omics datasets, indicating strong alignment between the multi-omics profiles as captured by the DIABLO model (**Figure 5B**). Heatmap based on DIABLO integrative analysis revealed distinct clustering and separation between HCC and HCC_M samples in terms of metabolite and protein expression profiles, suggesting that the two groups possess divergent molecular characteristics. Meanwhile, numerous metabolites and proteins exhibited concordant expression trends (i.e., simultaneous upregulation or downregulation) within the same sample group, indicating that they may be involved in identical metabolic pathways or regulatory networks (**Figure 5C**). Positive and negative correlations of significant magnitude were detected between proteins and metabolites (**Figure 5D**). A set of co-regulated features, which showed a strong association with the latent components of the multi-omics dataset, was identified and could be regarded as a characteristic molecular signature of HCC metastasis (**Figure 5E**). Functional enrichment analysis revealed that proteins in this co-regulated feature cluster were predominantly associated with the inflammatory metabolic reprogramming, oxidative stress response, gluconeogenesis, glycolysis, and TCA cycle (**Figure 5F**). The majority of metabolites were concentrated in amino acid metabolism, and typical examples included alanine, aspartate and glutamate metabolism, arginine and proline metabolism and glycerophospholipid metabolism, which plays a crucial role in inflammatory metabolic reprogramming, oxidative stress response and gluconeogenesis (**Figure 5G**). These findings suggest a potential regulatory crosstalk among inflammatory metabolic reprogramming, oxidative stress response, and amino acid metabolism, and this may serve as a promising approach to mitigate inflammatory metabolic dysfunction and oxidative stress during HCC metastasis.

### 3.6 Multivariable analysis of candidate biomarkers

To assess whether candidate biomarkers were independently associated with metastasis, multivariable logistic regression was performed adjusting for age, sex, AFP, HBsAg, ALT, AST, and GGT. CPS1 remained significantly associated with metastatic status (OR = 3.68, 95% CI: 1.24-10.87, P = 0.018), whereas PKM2 (OR = 1.70, P = 0.195) and L-glutamate (OR = 2.51, P = 0.278) were no longer significant after covariate adjustment (**Supplementary Figures 4**, **5**).

## 4 Discussion

HCC is characterized by high invasiveness and metastatic potential, and metastasis remains the leading cause of treatment failure and poor prognosis in HCC patients, which severely limits the long-term survival of patients (22, 23). Unraveling the pathophysiological features underlying HCC metastasis contributes to lowering its high morbidity and mortality rates. HCC metastasis has been found to be associated with metabolic reprogramming (24). Herein, we analyzed untargeted metabolomic profiles from blood specimens to uncover dynamic metabolic alterations throughout HCC metastasis. Based on the same batch of biological samples, we integrated multi-omics analysis of metabolomics and proteomics to build a systematic regulatory network spanning proteins and end-stage metabolites. This framework aids the biological interpretation of HCC metastasis and enables the identification of a unique biomarker signature defined by coordinated proteomic and metabolomic perturbations.

**Figure 6:**
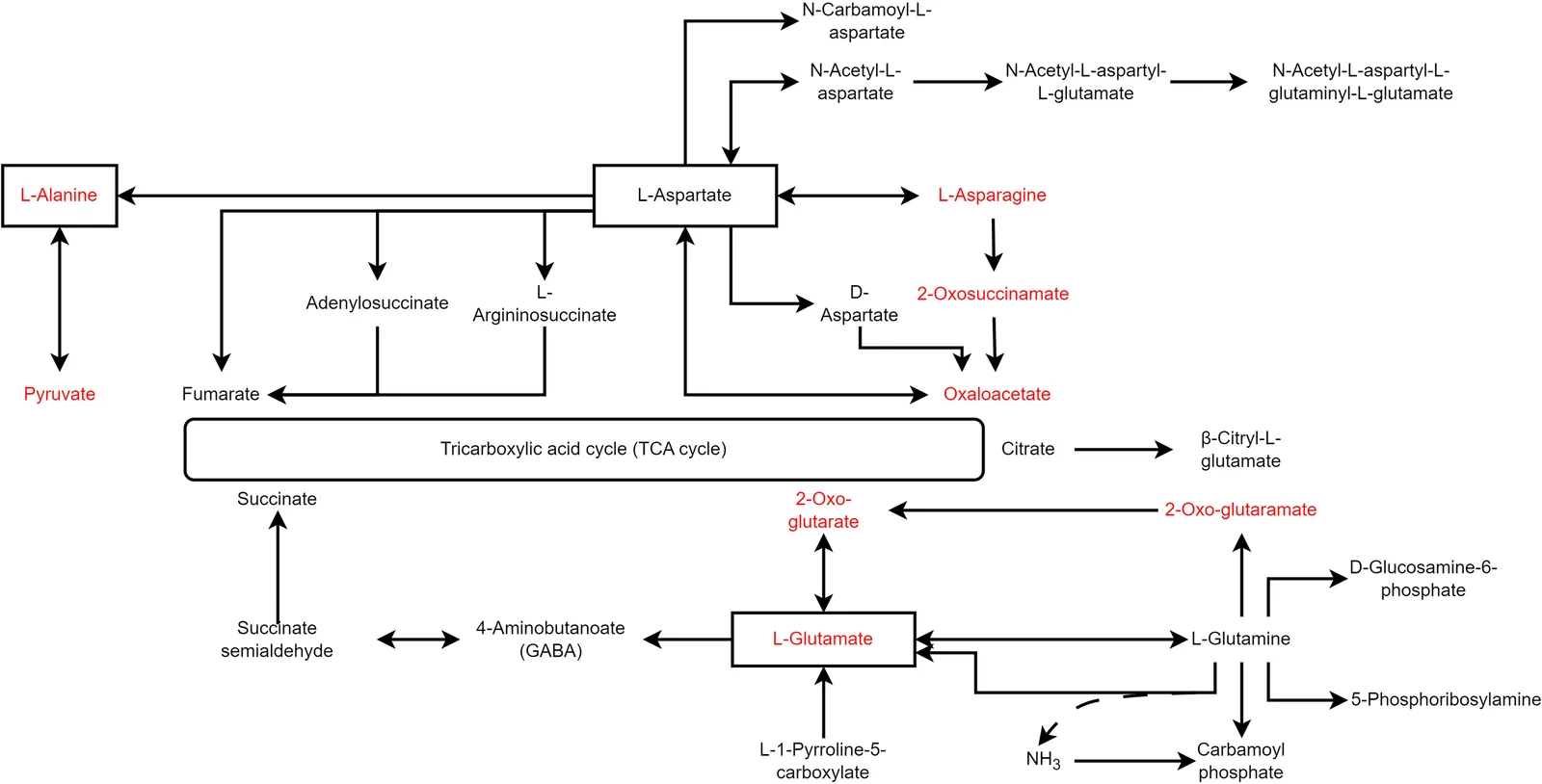
Schematic diagram of the crucial pathways. Alanine, aspartate and glutamate metabolism were the top hits from the pathway analysis of HCC_M-specific metabolites. The upregulated metabolites in HCC_M are colored red. HCC_M, HCC patients with metastasis.

Among all altered metabolic routes in HCC metastasis, amino acid metabolism—encompassing alanine, aspartate, glutamate, arginine-proline pathways and arginine biosynthesis—showed the most considerable and pervasive dysregulation. Previous studies have found that patients with metastatic HCC are accompanied by metabolic disorders characterized by changes in amino acid levels, which may be closely related to the pathogenesis of lung and brain metastases in HCC patients (25). Meanwhile, amino acid sensing has been confirmed to be associated with the regulation of inflammatory responses, oxidative stress, and apoptosis (26, 27). Currently, research on changes in amino acid consumption and their downstream molecular pathways has become an important field for exploring novel biomarkers and developing potential therapeutic targets (28). We found that essential intermediary metabolites involved in the alanine, aspartate and glutamate pathway were altered in HCC metastasis (**Figure 6**), with most intermediates, including L-alanine, pyruvate, L-asparagine, 2-oxosuccinamate, L-glutamate, 2-oxoglutarate, etc., exhibiting significant increases. Elevated levels of asparagine and 2-oxosuccinamate promote HCC cell invasion and migration during metastasis by modulating metabolic reprogramming and epithelial-mesenchymal transition (EMT) (29) and are thus closely linked to poor clinical outcomes in HCC patients (30). However, intracellular aspartic acid levels are significantly decreased during HCC metastasis. Reduced aspartic acid promotes EMT, invasion and migration by activating the PC-PLC/DAG/PKC signaling pathway, serving as a critical metabolic marker and independent adverse prognostic factor for HCC metastasis (31). During HCC metastasis, the levels of pyruvate and alanine are significantly elevated due to enhanced aerobic glycolysis (Warburg effect). High pyruvate promotes invasion and migration by inhibiting mitochondrial oxidation and activating EMT, while increased alanine provides biosynthetic substrates for metastatic cells (32). Moreover, HCC metastasis is accompanied by profound reprogramming of arginine and proline metabolism, characterized by downregulation of argininosuccinate synthase 1 (ASS1) and arginase 1 (ARG1) coupled with upregulation of transporters, leading to marked arginine accumulation that drives global metabolic reprogramming and promotes metastasis via EMT activation (33). Currently, multiple targeted agents, including sorafenib, lenvatinib, and donafenib, have been used clinically for HCC and have substantially improved the prognosis of patients with advanced disease (34). In contrast, metabolic-targeted therapies targeting proline (via PYCR1/P4HA1), arginine (via ADI-PEG20/ASS1), and glutamate (via CB-839/GLS1), although not yet approved for clinical application, have been validated in preclinical and phase I/II studies to significantly suppress EMT, invasion, and distant metastasis in HCC. Besides, these strategies exert synergistic antitumor effects in combination with immunotherapy or targeted therapy, representing a highly promising novel anti-metastatic approach (35).

In the present work, combined metabolomic and proteomic profiling of matched samples was applied to achieve systematic multi-omics integration. Such cross-omics integration enables the discovery of unique biological insights that cannot be captured by single-omics interrogation alone. Furthermore, identifying coordinated variable relationships across datasets derived from distinct methodological platforms and biological layers facilitates the construction of a robust, mutually validated molecular network. This framework allows systematic depiction of the biological cascade from protein-encoding genes to terminal metabolic outputs, thereby uncovering key regulatory drivers underlying complex disease pathogenesis, especially in cancer metabolic reprogramming (36). Moreover, these identified molecular signatures hold promise as candidate biomarkers for clinical diagnosis, as well as actionable targets for therapeutic intervention. Through integrative multi-omics analysis, twenty-five proteins (including PKM2, ALDH2 and GLS1)were identified as potential biomarkers for HCC metastasis, which is consistent with previously reported evidence (37–39)). Extracted from a multi-omics co-regulation network, these candidates occupy nodal positions in metastatic circuitry, supported by established mechanistic roles (e.g., PKM2 drives the Warburg effect; GLS1 governs glutaminolysis). However, their clinical translation is limited by the retrospective design and modest sample size (n = 105). Prospective multi-center validation across clinical stages and metastatic sites is warranted, and integration with established clinical indices may yield composite algorithms for early detection. Several of the identified metabolites (e.g., GLS1-mediated glutaminolysis intermediates, PKM2-driven glycolytic metabolites) have been independently validated in prior functional studies (40, 41). The high concordance between proteomic and metabolomic datasets in our analysis further supports the robustness of these findings. Analysis of functional enrichment for candidate proteins further clarified critical biological functions driving HCC metastasis, including the inflammatory response, oxidative stress, gluconeogenesis, and amino acid metabolism. Notably, the downstream metabolites regulated by these proteins were predominantly enriched in amino acid metabolism, including alanine, aspartate and glutamate pathway. These findings underscore that amino acid imbalance constitutes a major metabolic disorder in HCC metastasis.

WGCNA identified three functionally distinct metabolic-proteomic modules that collectively govern HCC metastasis through context-dependent network coordination. The green module, most strongly correlated with HCC metastasis (r = 0.75), represents a core metastatic engine driven by glutaminolysis-glycolysis coupling. Elevated L-glutamate and 2-oxoglutarate reflect GLS1/GLUD1-mediated glutamine catabolism, which replenishes the TCA cycle and generates biosynthetic precursors. Concurrently, PKM2 upregulation (identified in our proteomic analysis) diverts glycolytic flux toward lactate and anabolic intermediates, while GOT1 facilitates the malate-aspartate shuttle to maintain cytoplasmic NADPH pools (42). The salmon module, specific to HCC-associated lung metastasis (HCC_LM), reveals an aspartate-amide metabolic axis controlled by a feed-forward regulatory loop (43). CPS1 downregulation (DEP) impairs urea cycle flux, reducing ASS1-mediated argininosuccinate production and thereby sparing aspartate for ASNS-mediated asparagine synthesis, resulting in elevated asparagine levels (44), while elevated asparagine promotes epithelial-mesenchymal transition (EMT) (45), a process further linked to HCC metastatic potential. Conversely, the magenta module displayed a strong negative correlation with HCC-associated bone metastasis (HCC_BM) and was enriched in gluconeogenesis pathway, which enables tumor cells to synthesize glucose from non-carbohydrate precursors (lactate, glycerol, glucogenic amino acids) to maintain energy supply and biosynthetic demands under glucose limitation, enhancing adaptability to nutritional stress (46). Enrichment in gluconeogenesis indicates adaptive glucose production via PEPCK (DEP) under bone microenvironment glucose limitation, while tryptophan-kynurenine metabolism (potentially involving IDO1) generates both NAD^+^ and immunosuppressive metabolites (47, 48). Collectively, these findings highlight distinct metabolic signatures associated with different metastatic phenotypes in HCC. Importantly, these module-specific metabolic programs extend beyond cell-autonomous adaptations to actively remodel the tumor microenvironment (TME), thereby establishing distinct pro-metastatic niches.

Beyond cell-autonomous reprogramming, the metabolic enzymes identified within these modules actively reshape the tumor microenvironment (TME) to facilitate metastasis. The HCC TME is highly complex and etiology-dependent; a recent single-cell atlas revealed distinct immune-stromal compositions across HBV- and MASLD-driven HCC which impose divergent metabolic selective pressures (49). In this context, GLS1/GLUD1 activity in the green module generates glutamine-derived metabolites that polarize tumor-associated macrophages and potentiate STAT1-dependent PD-L1 expression, fostering immune evasion (50). Furthermore, altered amino acid metabolism may activate STAT3 to promote vascular remodeling at metastatic sites (51). These TME-directed metabolic programs are further reflected in systemic lipid dysregulation. Beyond amino acids, our analysis revealed that glycerophospholipids, arachidonic acid metabolites, and bile acids exhibited robust discriminatory power for HCC metastasis (high VIP scores and fold changes). Notably, glycerophospholipids were broadly downregulated in HCC_M patients, potentially enhancing membrane fluidity and invasive capacity (52). Conversely, arachidonic acid accumulation and its downstream eicosanoid shifts suggest a pro-inflammatory, pro-metastatic lipid milieu (53). Together, these lipid alterations may both drive metastatic behavior and serve as accessible circulating biomarkers. Collectively, these findings suggest that the metabolic reprogramming identified herein extends beyond tumor cells to establish a pro-metastatic ecosystem characterized by immune suppression, lipid dysregulation, and organ-specific adaptation. While our multi-omics data suggests metabolic-driven immune remodeling, direct experimental evidence linking the identified pathways to specific immune cell functions (e.g., T-cell exhaustion, macrophage M2 polarization, or MDSC recruitment) is lacking. Future studies integrating single-cell transcriptomics, multiplex immunohistochemistry, and *in vitro* co-culture assays are required to dissect the causal relationship between amino acid metabolic reprogramming and immune suppression in HCC metastasis. Several limitations should be acknowledged in this study. First, the derivation (n=70) and validation (n=35) cohorts were relatively small, and all specimens were retrospectively collected from a single center, which may limit generalizability, statistical power, and ethnic diversity. Serum-based profiling reflects systemic metabolism rather than the intratumoral microenvironment, potentially diluting organ-specific metabolic signatures. Stringent quality control and cross-omics profiling of identical specimens enhanced internal consistency, while robust methods (WGCNA and DIABLO) partially mitigated sample size concerns by leveraging variable correlations in high-dimensional data. Nevertheless, multi-center prospective validation with larger, ethnically diverse cohorts is essential. Second, OPLS-DA is inherently prone to overfitting; thus, the high AUROC values should be interpreted cautiously, as they may partly reflect the homogeneity of our single-center retrospective cohort. Although permutation testing and independent internal validation were applied, these measures cannot fully exclude center-specific bias. Larger prospective cohorts and multi-center studies with cross-site validation are therefore warranted to confirm the robustness and generalizability of these models. Third, untargeted LC–MS metabolomics is semi-quantitative and subject to ionization efficiency bias, while MS²-based annotation without authentic standard verification may yield unresolved isomers. Future studies should employ targeted MRM quantification with isotope-labeled standards. Fourth, metabolite annotation relied on a single commercial database; integration with public repositories (e.g., HMDB, KEGG, MassBank) is recommended for cross-validation. Fifth, while this study identified candidate biomarkers through rigorous multi-omics integration and independent cohort validation, we acknowledge that the findings are primarily hypothesis-generating. Experimental confirmation using orthogonal methods (e.g., ELISA, Western blotting, or immunohistochemistry) and functional assays (e.g., CRISPR-based gene editing, *in vitro* invasion assays and patient-derived xenograft models) are warranted to establish causal roles and clinical utility. Sixth, multivariable logistic regression adjusting for age, sex, AFP, HBsAg, ALT, AST, and GGT showed that CPS1 remained independently associated with metastasis (OR = 3.68, 95% CI: 1.24-10.87, P = 0.018), whereas PKM2 and L-glutamate lost significance. This suggests that alterations in PKM2 and L-glutamate may partly reflect liver dysfunction and tumor burden rather than metastasis-specific progression. Future studies using propensity-score matching or stage-stratified analyses are needed to distinguish metastasis-specific signatures from general cancer progression.

## 5 Conclusion

To conclude, integrated multi-omics analysis of metabolic and protein profiles was performed on matched samples from non-metastatic and metastatic HCC cohorts. Combined signature proteins and differential metabolites assemble into a holistic regulatory framework, which reflects the core metabolic dysregulation associated with HCC metastatic progression. Key biological mechanisms governing HCC progression were closely linked to amino acid metabolism, with prominent enrichment in alanine, aspartate and glutamate pathways. Additionally, the gluconeogenesis pathway was indicated to exert potential functional implications in HCC with brain metastasis. These dysregulated metabolites and proteins are promising candidates for developing novel diagnostic biomarkers and therapeutic targets for HCC metastasis. Future large-scale, multi-center prospective studies incorporating experimental validation are essential to confirm their diagnostic and therapeutic value.

## Data Availability

The original contributions presented in the study are included in the article/**Supplementary Material**. Further inquiries can be directed to the corresponding author/s.

## Supplementary material

Supplementary Table 1 Characteristics of enrolled patients with HCC included in the derivation group.

Supplementary Figure 1 Quality control (QC) of experimental data. **(A)** Comparison of the total ion chromatogram (TIC) of QC samples in positive ion mode (left) and negative ion mode (right). **(B)** Pearson correlation between QC samples in positive ion mode (left) and negative ion mode (right). **(C)** Principal component analysis (PCA) of the identified metabolites showing QC samples clustered together both in positive ion mode (left) and negative ion mode (right).

Supplementary Figure 2 Chance permutation at 200 times was used for the discrimination between HCC patients and HCC with metastasis patients in positive ion mode (left) and negative ion mode (right).

Supplementary Figure 3 Venn diagram showing the hub metabolites in positive ion mode (left) and negative ion mode (right).

Supplementary Figure 4 Multivariable logistic regression: biomarkers for HCC metastasis (adjusted for age, sex, AFP, HBsAg, ALT, AST, GGT).

Supplementary Figure 5 Multivariable logistic regression results (adjusted for age, sex, AFP, HBsAg, ALT, AST, GGT).

## References

[1] Rumgay H Arnold M Ferlay J Lesi O Cabasag CJ Vignat J . Global burden of primary liver cancer in 2020 and predictions to 2040. J Hepatol. (2022) 77:1598–606. doi: 10.1016/j.jhep.2022.08.021 36208844 PMC9670241

[2] Chan Y-T Zhang C Wu J Lu P Xu L Yuan H . Biomarkers for diagnosis and therapeutic options in hepatocellular carcinoma. Mol Cancer. (2024) 23:189. doi: 10.1186/s12943-024-02101-z 39242496 PMC11378508

[3] Forner A Reig M Bruix J . Hepatocellular carcinoma. Lancet. (2018) 391:1301–14. doi: 10.1016/s0140-6736(18)30010-2 29307467

[4] Galle PR Dufour J-F Peck-Radosavljevic M Trojan J Vogel A . Systemic therapy of advanced hepatocellular carcinoma. Future Oncol. (2020) 17:1237–51. doi: 10.2217/fon-2020-0758 33307782

[5] Liu G-M Zeng H-D Zhang C-Y Xu J-W . Identification of METTL3 as an adverse prognostic biomarker in hepatocellular carcinoma. Digestive Dis Sci. (2020) 66:1110–26. doi: 10.1007/s10620-020-06260-z 32333311

[6] Wang H Xu M Zhang T Pan J Li C Pan B . PYCR1 promotes liver cancer cell growth and metastasis by regulating IRS1 expression through lactylation modification. Clin Transl Med. (2024) 14:e70045. doi: 10.1002/ctm2.70045 39422696 PMC11488319

[7] Tzeng S-F Yu Y-R Park J von Renesse J Hsiao H-W Hsu C-H . PLT012, a humanized CD36-blocking antibody, is effective for unleashing antitumor immunity against liver cancer and liver metastasis. Cancer Discov. (2025) 15:1676–96. doi: 10.1158/2159-8290.Cd-24-1409 40294022 PMC7617665

[8] Ahmad P Moussa DG Siqueira WL . Metabolomics for dental caries diagnosis: past, present, and future. Mass Spectrom Rev. (2024) 44:454–90. doi: 10.1002/mas.21896 38940512

[9] Sun Y Zhang X Hang D Lau H-H Du J Liu C . Integrative plasma and fecal metabolomics identify functional metabolites in adenoma-colorectal cancer progression and as early diagnostic biomarkers. Cancer Cell. (2024) 42:1386–1400.e8. doi: 10.1016/j.ccell.2024.07.005 39137727

[10] Yang T Wang S-Y . Integrating transcriptome and metabolomics analyses of hepatocellular carcinoma to discover novel biomarkers and drug targets. Clinics Res Hepatol Gastroenterol. (2025) 49:102546. doi: 10.1016/j.clinre.2025.102546 39938636

[11] Zhang J Yang S Wang J Xu Y Zhao H Lei J . Integrated LC-MS metabolomics with dual derivatization for quantification of FFAs in fecal samples of hepatocellular carcinoma patients. J Lipid Res. (2021) 62:100143. doi: 10.1016/j.jlr.2021.100143 34710433 PMC8599149

[12] Liu J Geng W Sun H Liu C Huang F Cao J . Integrative metabolomic characterisation identifies altered portal vein serum metabolome contributing to human hepatocellular carcinoma. Gut. (2022) 71:1203–13. doi: 10.1136/gutjnl-2021-325189 34344785 PMC9120406

[13] Chen G Gou B Du Y Zhou Z Bai Y Yang Y . Predicting the molecular mechanism of ginger targeting PRMT1/BTG2 axis to inhibit gastric cancer based on WGCNA and machine algorithms. Phytomedicine. (2025) 143:156892. doi: 10.1016/j.phymed.2025.156892 40446574

[14] Yang C Chen X Liu J Wang W Sun L Xie Y . Identification and validation of pivotal genes in osteoarthritis combined with WGCNA analysis. J Inflammation Res. (2025) 18:1459–70. doi: 10.2147/jir.S504717 39906135 PMC11792882

[15] Barri T Dragsted LO . UPLC-ESI-QTOF/MS and multivariate data analysis for blood plasma and serum metabolomics: effect of experimental artefacts and anticoagulant. Anal Chim Acta. (2013) 768:118–28. doi: 10.1016/j.aca.2013.01.015 23473258

[16] Traub-Csekö YM Wong Y Rosa BA Becker L Camberis M LeGros G . Proteomic characterization and comparison of the infective and adult life stage secretomes from Necator americanus and Ancylostoma ceylanicum. PloS NeglTrop Dis. (2025) 19:e0012780. doi: 10.1371/journal.pntd.0012780 39832284 PMC11745416

[17] Lu C Huang X Huang M Liu C Xu J . Mendelian randomization of plasma proteomics identifies novel ALS-associated proteins and their GO enrichment and KEGG pathway analyses. BMC Neurol. (2025) 25:82. doi: 10.1186/s12883-025-04091-x 40033250 PMC11874834

[18] Zhao W Chen S Tian J Xue L Yang L Hu J . WGCNA-based integration of transcriptomics and metabolomics reveals key meat quality regulators during development in Jingyuan chickens. Poult Sci. (2025) 104:105843. doi: 10.1016/j.psj.2025.105843 40997594 PMC12495225

[19] Contreras N Escolar‐Peña A Delgado‐Dolset MI Fernández P Obeso D Izquierdo E . Multiomic integration analysis for monitoring severe asthma treated with mepolizumab or omalizumab. Allergy. (2024) 80:1899–911. doi: 10.1111/all.16434 39692160 PMC12261874

[20] Welham Z Déjean S KA LC . Multivariate analysis with the R package mixOmics. Methods Mol Biol. (2023) 2426:333–59. doi: 10.1007/978-1-0716-1967-4_15 36308696

[21] Rao G Sui J Zhang J . Metabolomics reveals significant variations in metabolites and correlations regarding the maturation of walnuts (Juglans regia L.). Biol Open. (2016) 5:829–36. doi: 10.1242/bio.017863 27215321 PMC4920193

[22] Pan J Zhang M Rao D Ma J Shen X Qin H . CAD manipulates tumor intrinsic DHO/UBE4B/NF-κB pathway and fuels macrophage cross-talk, promoting HCC metastasis. Hepatology. (2026) 83:451–65. doi: 10.1097/hep.0000000000001304 40073276

[23] Wei K Peng C Ou Y Wang P Zhan C Wei H . Decoding hepatocellular carcinoma metastasis: molecular mechanisms, targeted therapies, and potential biomarkers. Curr Issues Mol Biol. (2025) 47:263. doi: 10.3390/cimb47040263 40699663 PMC12025613

[24] Wang M Lyu H Liu Y Sun Y . Research progress on the mechanisms of traditional Chinese medicine in preventing and treating HCC invasion and metastasis based on lipid metabolic reprogramming. Curr Top Med Chem. (2025) 25:1502–20. doi: 10.2174/0115680266366687250730060318 40770475

[25] Wang Z Wu X Chen H-N Wang K . Amino acid metabolic reprogramming in tumor metastatic colonization. Front Oncol. (2023) 13:1123192. doi: 10.3389/fonc.2023.1123192 36998464 PMC10043324

[26] Xu K Shah P Makhanasa D Khan MW . Unveiling the maze: branched-chain amino acids fueling the dynamics of cancer metabolism and progression. Cancers. (2025) 17:1751. doi: 10.3390/cancers17111751 40507232 PMC12153561

[27] Duanmu Q Tan B Wang J Huang B Li J Kang M . The amino acids sensing and utilization in response to dietary aromatic amino acid supplementation in LPS-induced inflammation piglet model. Front Nutr. (2022) 8:819835. doi: 10.3389/fnut.2021.819835 35111801 PMC8801454

[28] Akinlalu A Ogberefor E Gao T Sun D . Targeting emerging amino acid dependencies and transporters in cancer therapy. Front Pharmacol. (2025) 16:1717414. doi: 10.3389/fphar.2025.1717414 41347155 PMC12672511

[29] Zhou Q Li L Sha F Lei Y Tian X Chen L . PTTG1 reprograms asparagine metabolism to promote hepatocellular carcinoma progression. Cancer Res. (2023) 83:2372–86. doi: 10.1158/0008-5472.Can-22-3561 37159932

[30] Bai J Tang R Zhou K Chang J Wang H Zhang Q . An asparagine metabolism-based classification reveals the metabolic and immune heterogeneity of hepatocellular carcinoma. BMC Med Genomics. (2022) 15:222. doi: 10.1186/s12920-022-01380-z 36284275 PMC9594908

[31] Chen S Tang Q Hu M Song S Wu X Zhou Y . Loss of carbamoyl phosphate synthetase 1 potentiates hepatocellular carcinoma metastasis by reducing aspartate level. Adv Sci. (2024) 11:e2402703. doi: 10.1002/advs.202402703 39387452 PMC11615744

[32] Montoya T Lee JV Qiu L Krall A Matulionis N Seo Y . Alanine catabolism as a targetable vulnerability for MYC-driven liver cancer. Biol Arch. (2025) 12:2025.07.29.667471. doi: 10.1101/2025.07.29.667471 41849351 PMC13264379

[33] Eun HS . Antitumor role of L-arginine and argininosuccinate synthetase 1 in hepatocellular carcinoma: direct and immunological mechanisms. J Liver Cancer. (2025) 25:1–3. doi: 10.17998/jlc.2025.03.07 40065673 PMC12010823

[34] Wang X Hu R Song Z Zhao H Pan Z Feng Y . Sorafenib combined with STAT3 knockdown triggers ER stress-induced HCC apoptosis and cGAS-STING-mediated anti-tumor immunity. Cancer Lett. (2022) 547:215880. doi: 10.1016/j.canlet.2022.215880 35981569

[35] Gao B Lu Y Lai X Xu X Gou S Yang Z . Metabolic reprogramming in hepatocellular carcinoma: mechanisms of immune evasion and therapeutic implications. Front Immunol. (2025) 16:1592837. doi: 10.3389/fimmu.2025.1592837 40370433 PMC12075234

[36] Dai Y-H Chang C-J Shen P-C Jheng W-L Lee D-J Chen Y-G . DriverOmicsNet: an integrated graph convolutional network for multi-omics exploration of cancer driver genes. Briefings Bioinf. (2025) 26:bbaf412. doi: 10.1093/bib/bbaf412 40814229 PMC12354958

[37] Zhou Q Yin Y Yu M Gao D Sun J Yang Z . GTPBP4 promotes hepatocellular carcinoma progression and metastasis via the PKM2 dependent glucose metabolism. Redox Biol. (2022) 56:102458. doi: 10.1016/j.redox.2022.102458 36116159 PMC9483790

[38] Qian J Huang C Wang M Liu Y Zhao Y Li M . Nuclear translocation of metabolic enzyme PKM2 participates in high glucose-promoted HCC metastasis by strengthening immunosuppressive environment. Redox Biol. (2024) 71:103103. doi: 10.1016/j.redox.2024.103103 38471282 PMC10945175

[39] Zhang H Fu L . The role of ALDH2 in tumorigenesis and tumor progression: targeting ALDH2 as a potential cancer treatment. Acta Pharm Sin B. (2021) 11:1400–11. doi: 10.1016/j.apsb.2021.02.008 34221859 PMC8245805

[40] Gao F Zhang X Wang S Zheng L Sun Y Wang G . TSP50 promotes the Warburg effect and hepatocyte proliferation via regulating PKM2 acetylation. Cell Death Dis. (2021) 12:517. doi: 10.1038/s41419-021-03782-w 34016961 PMC8138007

[41] Zhou Y Yu H Cheng S Chen Y He L Ren J . Glutamate dehydrogenase 1 mediated glutaminolysis sustains HCC cells survival under glucose deprivation. J Cancer. (2022) 13:1061–72. doi: 10.7150/jca.64195 35154470 PMC8824882

[42] Yang L Venneti S Nagrath D . Glutaminolysis: a hallmark of cancer metabolism. Annu Rev BioMed Eng. (2017) 19:163–94. doi: 10.1146/annurev-bioeng-071516-044546 28301735

[43] Zhang Z Du N He G Zhang M Zhang G Gao J . Lung cancer cell-intrinsic asparagine synthetase potentiates anti-tumor immunity via modulating immunogenicity and facilitating immune remodeling in metastatic tumor-draining lymph nodes. Int J Biol Sci. (2025) 21:6501–21. doi: 10.7150/ijbs.114791 41208878 PMC12594603

[44] Do LK Lee HM Ha YS Lee CH Kim J . Amino acids in cancer: understanding metabolic plasticity and divergence for better therapeutic approaches. Cell Rep. (2025) 44:115529. doi: 10.1016/j.celrep.2025.115529 40193251 PMC12038367

[45] Tan Z Boyapati K Tressler CM Jenkinson NM Glunde K . Glutamine transporter SLC38A3 promotes breast cancer metastasis via Gsk3β/β-catenin/EMT pathway. Cancer Lett. (2024) 586:216653. doi: 10.1016/j.canlet.2024.216653 38309615 PMC12832041

[46] Guo D Meng Y Zhao G Wu Q Lu Z . Moonlighting functions of glucose metabolic enzymes and metabolites in cancer. Nat Rev Cancer. (2025) 25:426–46. doi: 10.1038/s41568-025-00800-3 40175621

[47] Grasmann G Smolle E Olschewski H Leithner K . Gluconeogenesis in cancer cells - repurposing of a starvation-induced metabolic pathway? Biochim Biophys Acta Rev Cancer. (2019) 1872:24–36. doi: 10.1016/j.bbcan.2019.05.006 31152822 PMC6894939

[48] Anu RI Shiu KK Khan KH . The immunomodulatory role of IDO1-Kynurenine-NAD+ pathway in switching cold tumor microenvironment in PDAC. Front Oncol. (2023) 13:1142838. doi: 10.3389/fonc.2023.1142838 37456260 PMC10348419

[49] Zhang S Xu H Li M Yuan G Nie J Zhang Q . An etiology-stratified single-cell atlas identifies FABP4 as a prognostic marker for MASLD-related HCC. J Hepatol. (2026) 84:920–32. doi: 10.1016/j.jhep.2025.10.026 41205757

[50] Han H Gong C Zhang Y Liu C Wang Y Zhao D . RBM30 recruits DOT1L to activate STAT1 transcription and drive immune evasion in hepatocellular carcinoma. Oncogene. (2025) 44:3955–73. doi: 10.1038/s41388-025-03550-6 40883414

[51] Han H Yuan Y Li C Liu L Yu H Han G . RNA-binding motif protein 28 enhances angiogenesis by improving STAT3 translation in hepatocellular carcinoma. Cancer Lett. (2024) 604:217191. doi: 10.1016/j.canlet.2024.217191 39181434

[52] Li K Shi W Song Y Qin L Zang C Mei T . Reprogramming of lipid metabolism in hepatocellular carcinoma resulting in downregulation of phosphatidylcholines used as potential markers for diagnosis and prediction. Expert Rev Mol Diagn. (2023) 23:1015–26. doi: 10.1080/14737159.2023.2254884 37672012

[53] Yan Y-C Meng G-X Yang C-C Yang Y-F Tan S-Y Yan L-J . Diacylglycerol lipase alpha promotes hepatocellular carcinoma progression and induces lenvatinib resistance by enhancing YAP activity. Cell Death Dis. (2023) 14:404. doi: 10.1038/s41419-023-05919-5 37414748 PMC10325985

