# Supplementary material for: Integrated multi-omics analysis of metabolomics and proteomics uncovers dysregulated amino acid metabolism in HCC metastasis
Source: Front Immunol. 2026 Aug 19;17:1856643. doi: 10.3389/fimmu.2026.1856643 (PMC13534100; doi:10.3389/fimmu.2026.1856643)
Supplement: Supplementary file 1 [file Table1.docx]

**Table S1 Characteristics of enrolled patients with HCC included in the derivation group**

| **Characteristic** | **HCC** | **HCC_M** | ***P* value** |
| --- | --- | --- | --- |
| n | 30 | 40 |  |
| Male (%) | 28 (93.3%) | 37 (92.5%) | 0.893 |
| Age (mean ± SD) | 60.5 ± 11.0 | 57.2 ± 10.1 | 0.400 |
| Total Protein (g/L) | 65.9 ± 6.9 | 67.9 ± 7.9 | >0.05 |
| Albumin (g/L) | 36.1 ± 4.3 | 38.2 ± 5.9 | >0.05 |
| Globulin (g/L) | 29.9 ± 6.0 | 29.4 ± 7.7 | >0.05 |
| Albumin/Globulin Ratio | 1.3 ± 0.3 | 1.5 ± 1.0 | >0.05 |
| Alanine aminotransferase (U/L) | 23.5 ± 16.6 | 35.8 ± 24.3 | >0.05 |
| Aspartate aminotransferase (U/L) | 33.3 ± 14.8 | 54.0 ± 23.5 | 0.001 |
| mitochondrial AST (U/L) | 4.5 ± 2.6 | 18.4 ± 14.9 | 0.000 |
| Alkaline phosphatase (U/L) | 96.3 ± 33.9 | 119.5 ± 63.9 | 0.004 |
| GGT (U/L) | 61.2 ± 54.1 | 88.4 ± 76.4 | 0.004 |
| TBIL (μmol/L) | 22.6 ± 12.6 | 19.9 ± 11.8 | >0.05 |
| DBIL (μmol/L) | 3.8 ± 3.4 | 4.0 ± 3.1 | >0.05 |
| IBIL (μmol/L) | 18.8 ± 9.8 | 16.3 ± 7.7 | >0.05 |

Data are expressed as the mean ± SD. Continuous variables were compared by using Student’s t test.

AST=Aspartate Aminotransferase. GGT=Gamma-Glutamyl Transferase. TBIL=Total Bilirubin. DBIL

=Direct Bilirubin. IBIL= Indirect Bilirubin. FDR-adjusted P < 0.05.
